# Coordination of the canonical and noncanonical IKK/NF-κB signaling pathways in HTLV-I Tax-mediated tumorigenesis

**DOI:** 10.1186/1742-4690-8-S1-A157

**Published:** 2011-06-06

**Authors:** Jing Fu, Zhaoxia Qu, Pengrong Yan, Shirong Li, Xinxin Song, Chie Ishikawa, Rami I Aqeilan, Naoki Mori, Arnold B Rabson, Gutian Xiao

**Affiliations:** 1 University of Pittsburgh Cancer Institute, Department of Microbiology and Molecular Genetics, University of Pittsburgh School of Medicine, Pittsburgh, PA, USA; 2 Transdisciplinary Research Organization for Subtropics and Island Studies, University of the Ryukyus, Okinawa, Japan; 3 The Lautenberg Center for General and Tumor Immunology, Institute for Medical Research Israel-Canada, Hebrew University-Hadassah Medical School, Jerusalem, Israel; 4 Child Health Institute of New Jersey, Departments of Pediatrics, Molecular Genetic, Microbiology and Immunology, UNDNJ-RWJMS, New Brunswick, NJ, USA

## 

Although the mechanisms by which the HTLV-I Tax oncoprotein activates the two NF-κB signaling pathways have been well studied, there is still no convincing evidence demonstrating a functional role for NF-κB in the Tax-mediated tumorigenesis or HTLV-I-mediated adult T-cell leukemia/lymphoma (ATL). Moreover, how Tax is negatively regulated by cellular factors has not yet been explored. Using various transgenic mice and cells deficient in different key IKK/NF-κB signaling components, we have defined the differential but cooperative roles of the canonical and noncanonical NF-κB pathways in Tax-mediated tumorigenesis. One function of non-canonical NF-κB activation is to repress expression of the wwox tumor suppressor gene, which in turn facilitates Tax-induced canonical NF-κB activation. Mechanistic studies indicate that WWOX blocks Tax-induced IKKalpha recruitment and subsequent RelA phosphorylation. Furthermore, we have identified PDLIM2, an essential terminator of canonical NF-κB activation, as a negative regulator of Tax. PDLIM2 directly binds to and shuttles Tax from its activation sites to the nuclear matrix for ubiquitination-mediated degradation, thereby suppressing the tumorigenicities of HTLV-I- or Tax-transduced cells. Interestingly, PDLIM2 expression is epigenetically downregulated in HTLV-I-transformed T cells and primary ATL cells. These studies suggest that the counterbalance between PDLIM2 and HTLV-I may determine the ATL leukemogenesis and provide the first line of genetic evidence demonstrating the functional significance of NF-κB in Tax-mediated tumorigenesis. These studies also define, for the first time, a delicate cooperation of the two pro-oncogenic NF-κB pathways in tumorigenesis and a functional role of the WWOX tumor suppressor in NF-κB regulation and viral tumorigenesis.

